# Supplementary material for: Sequential Extraction of Bioactive Saponins from Cucumaria frondosa Viscera: Supercritical CO2–Ethanol Synergy for Enhanced Yields and Antioxidant Performance
Source: Mar Drugs. 2025 Jun 28;23(7):272. doi: 10.3390/md23070272 (PMC12299899; doi:10.3390/md23070272)
Supplement: Supplementary file 1 [file marinedrugs-23-00272-s001.zip › marinedrugs-3733611-supplementary.pdf]

Supporting Information

Sequential Extraction of Bioactive Saponins from *Cucumaria frondosa* Viscera: Supercritical CO_2–Ethanol Synergy for Enhanced Yields and Antioxidant Performance

Jianan Lin [1], Guangling Jiao [2] and Azadeh Kermanshahi-pour [1,*]

[1] Biorefining and Remediation Laboratory, Department of Process Engineering and Applied Science, Dalhousie University, Halifax, NS B3J 1B6, Canada; jianan.lin@dal.ca
[2] AKSO Marine Biotech Inc., Hacketts Cove, NS B3Z 3K7, Canada;
* Correspondence: azadeh.kermanshahipour@dal.ca

Table S1. The preliminary one-single-factor experiments for subsequent $scCO_2$ extraction of saponins from *C. frondosa* viscera (mg OAE/g of samples on a dry weight basis, n=2, mean±SD).

Trial	Subsequent saponin extraction [1]		Saponin yields (mg OAE/g) [2]		
	Static extraction time	EtOH co-solvents	1st	2nd	Total
1	10 min	3.75 mL in the static, none in the dynamic	1.11±0.29	3.19±0.29	4.30±0.11
2	30 min	3.75 mL in the static, 7.5 mL in the dynamic at 0.5 mL/min	0.74±0.03	3.21±1.18	3.94±1.21
3	10 min	3.75 mL in the static, 7.5 mL in the dynamic at 0.5 mL/min	0.70±0.02	3.84±1.05	4.54±1.07
4	10 min	3.75 mL in the static, continuous feeding in the dynamic at 0.5 mL/min	0.63±0.01	5.99±1.32	6.63±1.33
5	10 min	None in the static, continuous feeding in the dynamic at 0.5 mL/min	1.50±0.20	5.07±0.50	6.56±0.65
6	10 min	7.5 mL in the static, continuous feeding in the dynamic at 0.5 mL/min	1.33±0.27	5.41±0.49	6.74±0.21

[1] Before subsequent saponin $scCO_2$ extraction, lipid $scCO_2$ extraction was performed under the optimal conditions (75 °C, 45 MPa, 20 min static, 30 min dynamic extraction, and 2:1 of

95% EtOH to feedstock mass ratio) obtained from the previous work [1]. Besides static extraction times and co-solvent loading-related parameters, other conditions were fixed: 55 ˚C, 35 MPa, 45 min of dynamic extraction, and 75% EtOH used as co-solvent.

[2] 1st and 2nd refer to saponin yields in the step of $scCO_2$ extraction of lipids and the subsequent step of $scCO_2$ extraction of saponins, and the total is the combination of 1st and 2nd.

Table S2. The definitive screening design and additional single-factor experiments for subsequent $scCO_2$ extraction of saponins from *C. frondosa* viscera (mg OAE/g of samples on a dry weight basis; n=2, mean±SD if applicable).

# [1]	Temp (˚C)	Pres (MPa)	SET (min)	Vol (mL)	DET (min)	Conc (%)	Saponin yields (mg OAE/g) [2]		
							1st	2nd	Total
1	35	20	15	0	90	100	1.70	2.06	3.76
2	75	20	0	7.5	60	100	1.37	3.87	5.25
3	35	50	30	0	60	50	2.82	3.36	6.18
4	75	50	15	7.5	30	50	3.80	3.95	7.75
5	75	20	30	3.75	90	50	5.13	6.47	11.61
6	35	50	0	3.75	30	100	4.61	7.06	11.67
7	75	50	0	0	90	75	3.38	4.40	7.78
8	75	35	30	0	30	100	2.30	1.74	4.04
9	55	50	30	7.5	90	100	2.69	6.67	9.36
10	55	20	0	0	30	50	2.87	3.65	6.52
11	35	35	0	7.5	90	50	3.54	5.16	8.70
12	55	35	15	3.75	60	75	1.59	5.70	7.28
13	35	20	30	7.5	30	75	2.71	5.78	8.50
A1	75	50	15	3.75	60	75	3.63±0.66	6.60±0.18	10.23±0.84
A2	35	50	15	3.75	60	75	2.19±0.50	6.07±0.28	8.26±0.22
A3	35	20	15	3.75	60	75	2.10±0.56	6.78±1.36	8.88±1.92

[1] 1-13 stands for the runs resulting from the definitive screening design, while A1-3 represents additional single-factor experiments exploring the impacts of the two main $scCO_2$ system criteria, temperature and pressure.

[2] 1st and 2nd refer to saponin yields in the step of $scCO_2$ extraction of lipids and the subsequent step of $scCO_2$ extraction of saponins, and the total is the combination of 1st and 2nd.

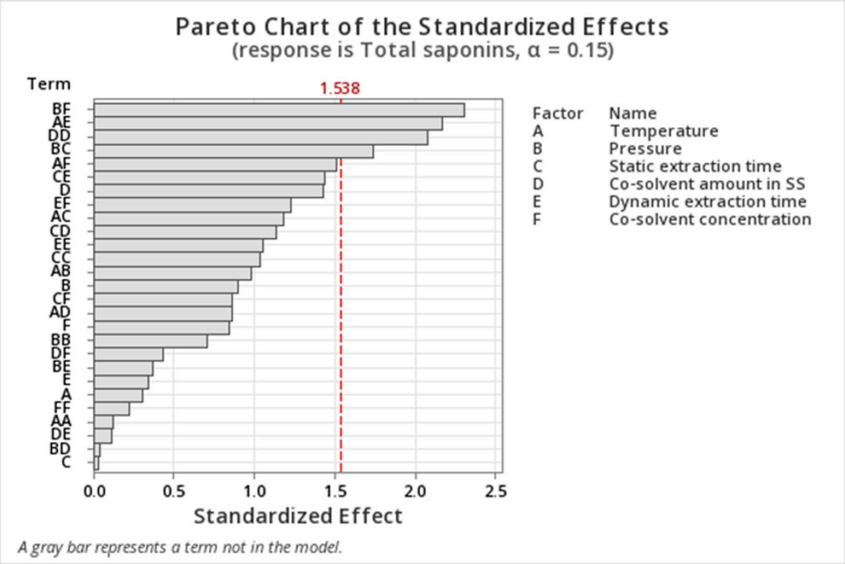

Stepwise Selection of Terms

Candidate terms: Temperature, Pressure, Static extraction time, Co-solvent amount in SS, Dynamic extraction time, Co-solvent concentration, Temperature*Temperature, Pressure*Pressure, Static extraction time*Static extraction time, Co-solvent amount in SS*Co-solvent amount in SS, Dynamic extraction time*Dynamic extraction time, Co-solvent concentration*Co-solvent concentration, Temperature*Pressure, Temperature*Static extraction time, Temperature*Co-solvent amount in SS, Temperature*Dynamic extraction time, Temperature*Co-solvent concentration, Pressure*Static extraction time, Pressure*Co-solvent amount in SS, Pressure*Dynamic extraction time, Pressure*Co-solvent concentration, Static extraction time*Co-solvent amount in SS, Static extraction time*Dynamic extraction time, Static extraction time*Co-solvent concentration, Co-solvent amount in SS*Dynamic extraction time, Co-solvent amount in SS*Co-solvent concentration, Dynamic extraction time*Co-solvent concentration

α to enter = 0.15, α to remove = 0.15

No terms can enter the model.

(1) Alpha = 0.15

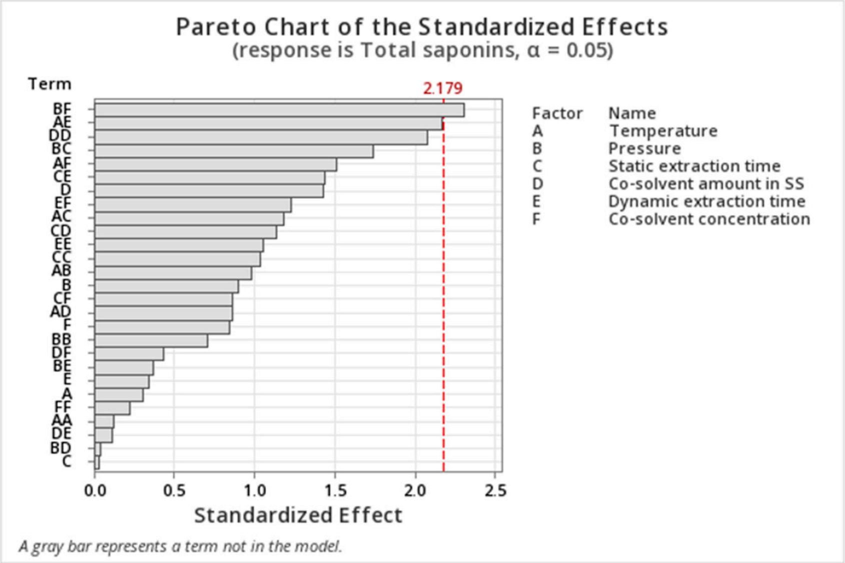

Stepwise Selection of Terms

Candidate terms: Temperature, Pressure, Static extraction time, Co-solvent amount in SS, Dynamic extraction time, Co-solvent concentration, Temperature*Temperature, Pressure*Pressure, Static extraction time*Static extraction time, Co-solvent amount in SS*Co-solvent amount in SS, Dynamic extraction time*Dynamic extraction time, Co-solvent concentration*Co-solvent concentration, Temperature*Pressure, Temperature*Static extraction time, Temperature*Co-solvent amount in SS, Temperature*Dynamic extraction time, Temperature*Co-solvent concentration, Pressure*Static extraction time, Pressure*Co-solvent amount in SS, Pressure*Dynamic extraction time, Pressure*Co-solvent concentration, Static extraction time*Co-solvent amount in SS, Static extraction time*Dynamic extraction time, Static extraction time*Co-solvent concentration, Co-solvent amount in SS*Dynamic extraction time, Co-solvent amount in SS*Co-solvent concentration, Dynamic extraction time*Co-solvent concentration

α to enter = 0.05, α to remove = 0.05

No terms can enter the model.

(2) Alpha = 0.05

Figure S1. Pareto chart of the standardized effects for total saponin yields. This Pareto chart illustrates the relative magnitude and statistical significance of standardized effects from the definitive screening design used to evaluate total saponin yield. Standardized effects are plotted in decreasing order of their absolute values, allowing for visual comparison of variable importance. All bars are gray, indicating that no terms met the criteria for entry into the model. This suggests that none of the main, interaction, or quadratic effects significantly influenced the total saponin yield within the tested ranges.

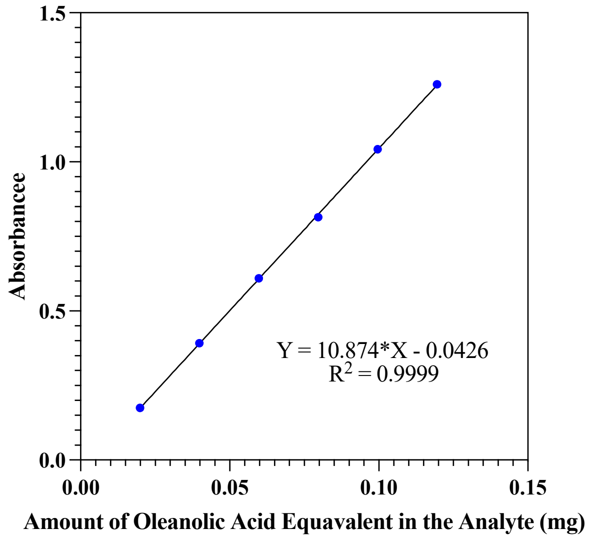

Figure S2. The standard curve of the amount of OAE as the X-axis and the absorbance as the Y-axis.
